# Attitudes, weight stigma and misperceptions of weight loss strategies among patients living with obesity in the Lazio Region, Italy

**DOI:** 10.3389/fendo.2024.1434360

**Published:** 2024-07-15

**Authors:** Luca Colangeli, Benedetta Russo, Esmeralda Capristo, Stefania Mariani, Dario Tuccinardi, Melania Manco, Valeria Scipione, Maria Eugenia Parrotta, Danila Capoccia, Valeria Guglielmi

**Affiliations:** ^1^ Department of Systems Medicine, University of Rome Tor Vergata, Rome, Italy; ^2^ Internal Medicine Unit - Obesity Center, University Hospital Policlinico Tor Vergata, Rome, Italy; ^3^ Unit of Endocrinology, Isola Tiberina - Gemelli Isola Hospital, Rome, Italy; ^4^ Executive Committee of the Regional Section of the Italian Obesity Society (SIO Lazio), Rome, Italy; ^5^ Department of Medical and Surgical Sciences, Fondazione Policlinico Universitario A. Gemelli IRCSS, Rome, Italy; ^6^ Department of Translational Medicine and Surgery, Catholic University of the Sacred Heart, Rome, Italy; ^7^ Department of Experimental Medicine, Section of Medical Pathophysiology, Food Science and Endocrinology, Policlinico Umberto I, Sapienza University of Rome, Rome, Italy; ^8^ Department of Endocrinology and Diabetes, University Campus Bio-Medico of Rome, Rome, Italy; ^9^ Preventive and Predictive Medicine Unit, Bambino Gesù Children’s Hospital, IRCCS, Rome, Italy; ^10^ Department of Medical Surgical Sciences and Biotechnologies, Sapienza University of Rome, Santa Maria Goretti Hospital, Latina, Italy

**Keywords:** obesity, survey, patients, stigma, anti-obesity medications, misperceptions, educational needs

## Abstract

**Introduction:**

Patient engagement is essential to achieve long-term goals in obesity management. It is crucial to identify patients’ perspectives, misperceptions and unmet educational needs on obesity etiology and treatments, to establish a correct therapeutic alliance between healthcare providers and patients.

**Methods:**

Objective: This study, promoted by the regional section of the Italian Obesity Society (SIO Lazio), explores attitudes towards obesity, self-awareness, misperceptions of weight loss strategies, experiences of weight stigma and educational needs of patients living with overweight or obesity. Design and subject: We conducted an anonymous survey among patients who accessed an Obesity Management Centers across the Lazio region of Italy for the first time, from October 2023 to April 2024. Approach: The survey consisted of 27 closed-ended questions grouped into 4 sections: (1) sociodemographic factors and self-reported anthropometric measures; (2) self-awareness and attitudes towards obesity and weight loss strategies; (3) experiences of obesity-related stigma; (4) knowledge and perceptions of obesity treatment options.

**Results:**

A total of 300 patients (67.9% women, aged 49.1 ± 14.4 years) returned completed surveys. Despite the self-reported BMI 35.3 ± 7.0 kg/m^2^ with three out of four (75.3%) of participants having a BMI ≥ 30 kg/m^2^, only 49% correctly identified themselves as affected by obesity. Almost one-third of the patients believed that obesity does not imply a genetic predisposition (31.9%) and that it is always secondary to psychological or behavioral disorders (29.7%). Interestingly, 66.7% of the patients declared themselves as completely responsible for their own condition and 39.4% considered obesity always treatable by means of lifestyle interventions. Stigma and weight discrimination in healthcare settings were reported by a substantial portion of patients (31.9%). A perception of inadequate support from the National Healthcare System emerged in most of the interviews (61.9%). Most patients (72.1%) felt they were not sufficiently informed about anti-obesity medications and a relevant part of their knowledge was derived from healthcare providers (57.7%) and social networks (19.1%). Weight loss medications were considered useful (63.2%) or necessary (18.4%) by the majority of patients, but only 60.1% would accept without any hesitation a pharmacologic treatment. The main reasons for refusal of pharmacological treatments were the belief that lifestyle intervention is a sufficient treatment (46.9%), the fear of adverse effects (28.1%) and feeling defeated (12.5%). Similarly, for most of participants bariatric surgery is useful (73.3%) or necessary (13.6%).

**Conclusion:**

Despite advancements in obesity research, this study underscores the need to improve patient education and public awareness to optimize the management and treatment of obesity. Addressing misconceptions, stigma, and gaps in knowledge are critical steps towards improving patient outcomes and fostering a supportive healthcare environment.

## 1 Introduction

Obesity is a chronic progressive, relapsing and symptomatic disease (1) that has reached epidemic proportions worldwide and is primarily caused by people’s latent genetic and biological susceptibility interacting with a changing obesogenic environment (2). Obesity is associated with an increased risk of morbidity, disability, lower life expectancy, reduced quality of life and increased direct and indirect healthcare costs (3). Furthermore, people with obesity face stigma, negative attitudes, prejudice and social discrimination in many settings like schools, workplaces and healthcare facilities. Obesity stigma not only results in negative psychosocial outcomes (4), but also deters people with obesity from engaging in healthy lifestyle and seeking healthcare services, thus compromising their overall healthcare (5–7).

Recognition of obesity as a disease is a public process that entails a deeper knowledge of the genetic, metabolic, environmental and social factors causing obesity far beyond personal choice, increased awareness of costs and expectations of benefits (3). However, the general public has been slow to reframe obesity as a disease, rather than a reversible condition due to unhealthy choices that reflect ignorance or lack of motivation, so that obesity still remains largely biased, underdiagnosed and undertreated. Therefore, there is an urgent need to identify local barriers, unmet educational needs on obesity etiology and misperceptions of the safety and/or efficacy of bariatric surgery and currently available weight loss medications among healthcare professionals and patients to build a positive and constructive therapeutic alliance and achieve better long-term outcomes (8).

In a previous survey study questioning primary care practitioners (PCPs) demonstrated some understanding of the complex nature of obesity and adopted a correct approach assessing patients with obesity on the first visit, scarce familiarity with indications for treatment and referrals and therapeutic inertia emerged (9). In the present study, we aimed to identify attitudes towards obesity- and health-related risks, self-awareness, perceptions of weight loss strategies, experiences of obesity-related stigma and educational needs of patients living with overweight or obesity who accessed for the first time an Obesity Management Centre across the Lazio region, central Italy.

## 2 Methods

### 2.1 Study design and sample

We conducted an anonymous survey study from October 2023 to April 2024 to investigate attitudes towards obesity, self-awareness, misperceptions of weight loss strategies, experiences of obesity-related stigma and educational needs of patients living with overweight or obesity who accessed for the first time an Obesity Management Centre across the Lazio region, with most of the centers being accredited under the EASO’s Collaborating Centers for Obesity management initiative.

The survey was proposed to all patients on a voluntary basis. The online version of the survey was created using the “Google Survey^®^” platform that patients could access by scanning a QR code with their smartphone. Alternatively, patients could fill out a paper questionnaire, subsequently transcribed online by a researcher. The study was promoted by the regional section of the Italian Obesity Society (SIO Lazio).

The study was conducted according to the requirements of the Declaration of Helsinki and the data collected were processed according to EU Regulation No. 2016/679 (GDPR), Legislative Decree No.196/2003 “Code on the Protection of Personal Data” and the subsequent amendments, and all the current legislation on data processing and protection. No information that could possibly render responders identifiable was collected. In Italy, ethical approval was determined to be non-essential for a study of anonymous nature, based on regulatory standards and precedent (10). The study complied with all laws and regulations regarding the management of personal information as required by Italy and the European General Data Protection Regulation.

### 2.2 Questionnaire

The survey consisted of 27 multiple choice questions grouped into 4 sections: (1) sociodemographic factors; (2) self-awareness and attitudes towards obesity and lifestyle intervention; (3) experiences of weight stigma; (4) knowledge and perceptions of obesity treatment options. No information that could render the data subject identifiable was collected. Only participants with completed questionnaires were included in the analyses.

### 2.3 Statistical analysis

Descriptive statistics were obtained for all study variables. Categorical variables were summarized as counts and percentages. Statistical analysis was conducted using IBM SPSS Statistics (IBM SPSS Statistics for Windows, Version 28.0. Armonk, NY: IBM Corp.).

## 3 Results

### 3.1 Participants

A total of 300 patients returned completed surveys. Of these, 55% had been accessing for the first time the outpatient services of University Hospital Policlinico Tor Vergata of Rome, 28.9% Isola Tiberina – Gemelli Isola of Rome, 8.1% University Hospital Policlinico Agostino Gemelli of Rome, 1.3% University Hospital Campus Biomedico of Rome, and 6.7% Santa Maria Goretti Hospital of Latina. The sample had a mean age of 49.1 ± 14.4 years (range = 18–79) and 67.9% were women.

Almost half (44.4%) of the participants had been referred by physicians (specialists: 32.9%; primary care practitioners: 11.5%), while the others came to visit as their own decision (29.2%) or followed the suggestion of relatives (12.9%) and acquaintances (13.6%).

**Figure 1:**
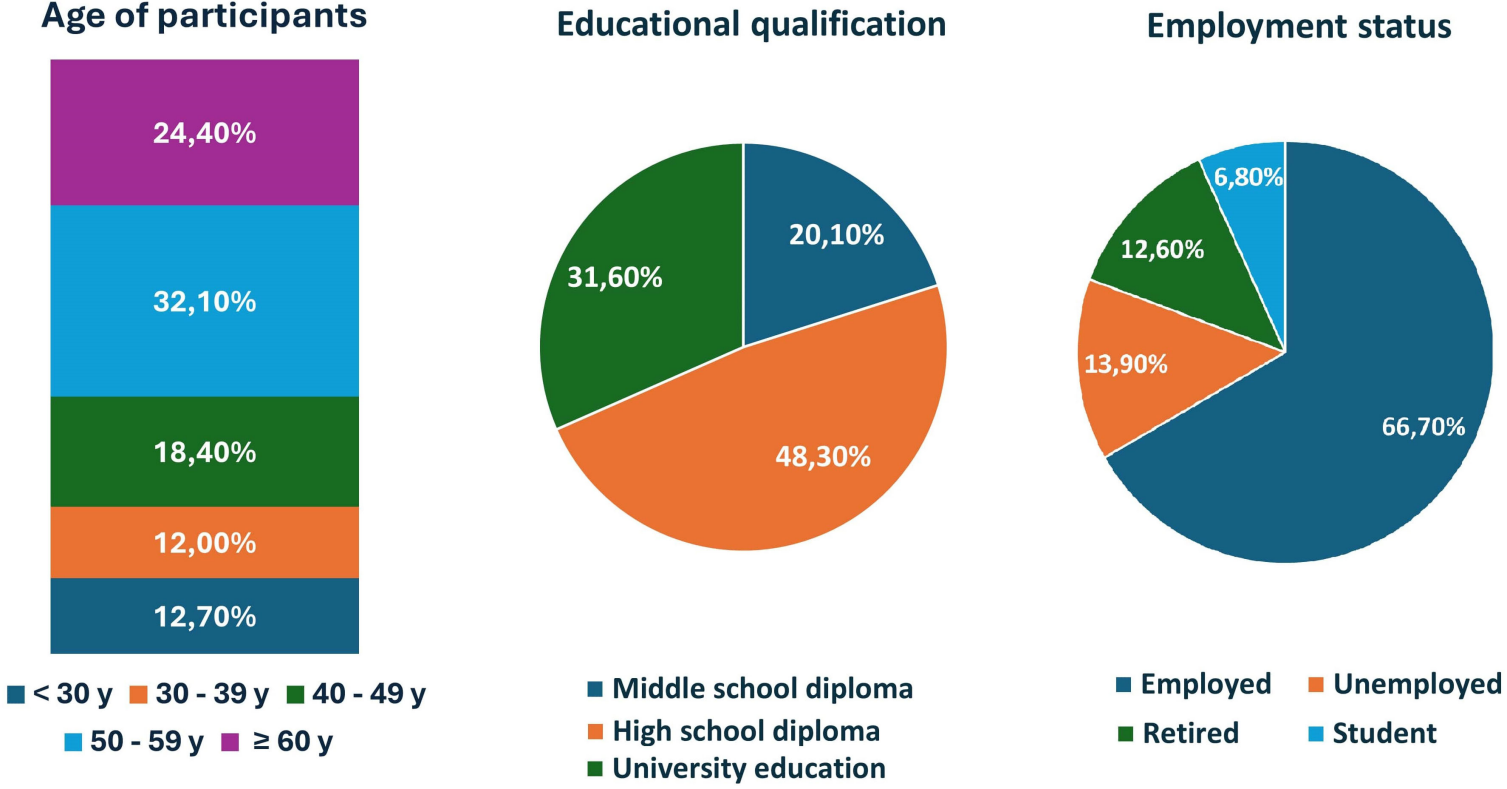
Participants’ distribution according to age category, educational qualification and employment position.

The majority lived in Rome (84.2%), while the remainders were from the provinces of Latina (8.1%), Frosinone (2.7%), Viterbo (0.7%), or lived outside the Lazio region (4.4%). One out of three participants had an academic degree (31.6%), while the others had completed high school (48.3%) or had education below high school level (20.1%). With regard to the employment status, over the half (66.7%) had a job, while the others were unemployed (13.9%), retired (12.6%) or students (6.8%) (**Figure 1**).

### 3.2 Self-awareness and attitudes towards obesity and lifestyle interventions

**Figure 2:**
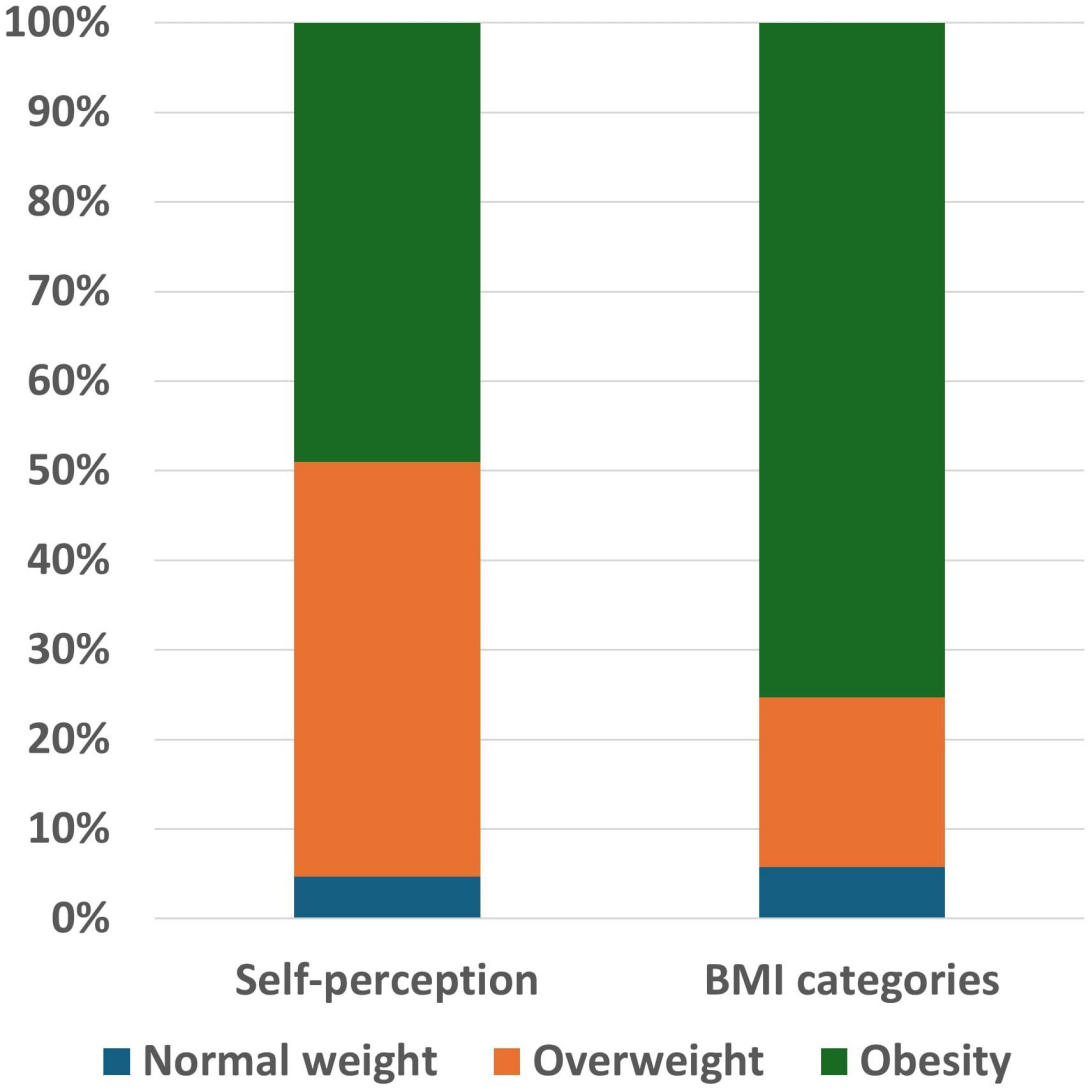
Class of weight distribution according to self-perception and to BMI categories.

Despite the self-reported BMI 35.3 ± 7.0 kg/m^2^ with three out of four (75.3%) of participants having a BMI ≥ 30 kg/m^2^, only 49% correctly identified themselves as affected by obesity. The remaining 46.3% and 4.7% defined themselves as overweight or normal weight, respectively (**Figure 2**). The reported duration of overweight/obesity was longer than 5 years for over the half of subjects (55.5%) and since infancy for 24.7%.

The majority reported having at least one first degree relative affected by obesity (68.8%) and considered obesity as a hereditary (22.4%) or a partially hereditary disease (45.8%).

**Figure 3:**
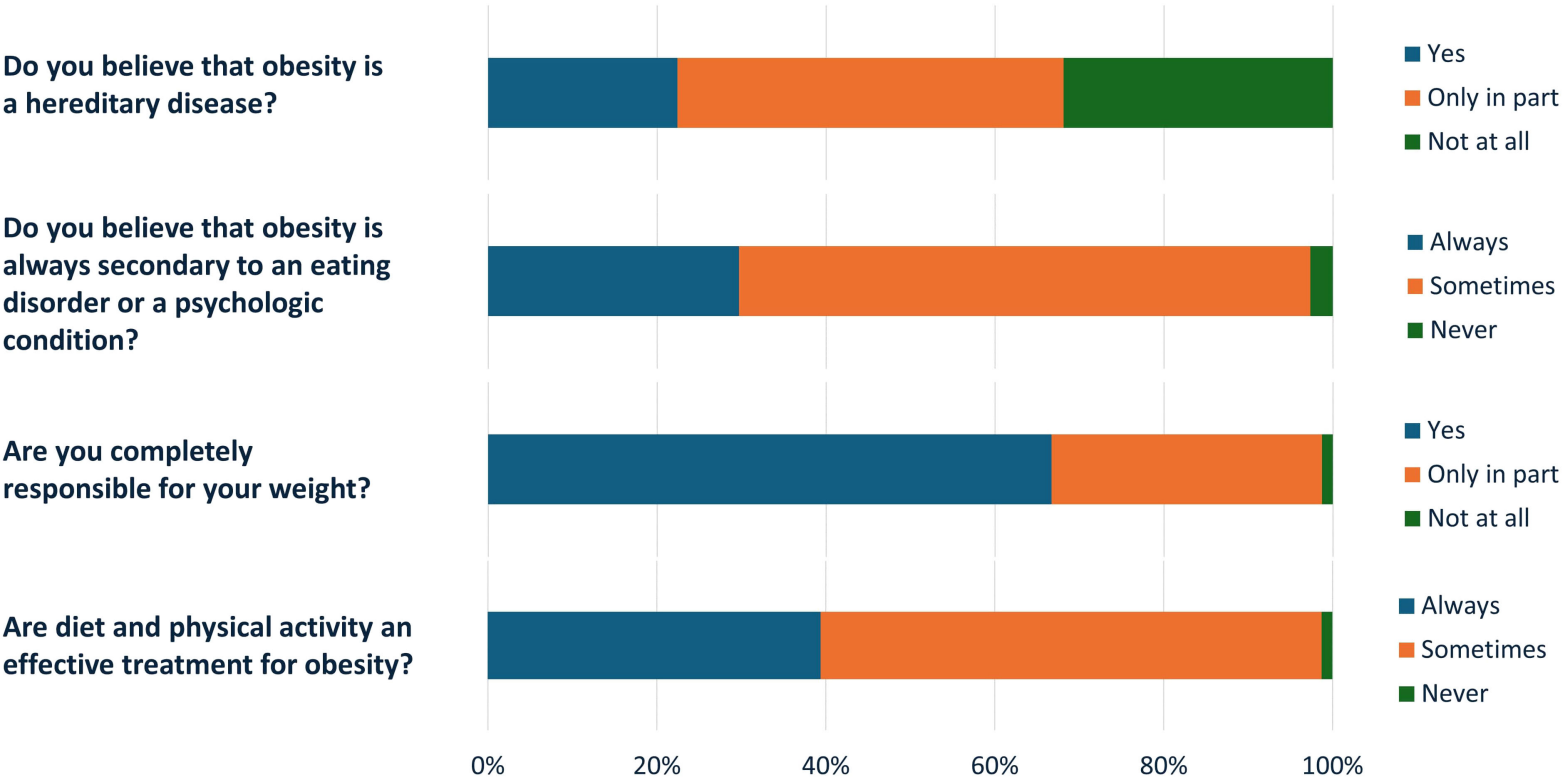
Patients’ beliefs on obesity cause and weight loss strategies.

However, almost one third of the patients believed that obesity does not imply a genetic predisposition (31.9%) (**Figure 3**). Most (67.6%) considered obesity at least in part associated to nutritional or psychological issues and for 29.7% of participants it is always secondary to a psychologic or behavioral disorders (**Figure 3**).

Interestingly, 66.7% of the patients declared themselves as completely responsible for their own condition and 32% only partially responsible. Accordingly, 39.4% considered obesity always treatable by means of lifestyle interventions (diet and physical activity) (**Figure 3**).

For almost half (42.4%) of the participants the most appropriate specialists for the obesity management and treatment are obesiologists (physicians specialized in the treatment of obesity), followed by nutritionists (33.2%), dieticians (10.5%), primary care physicians (9.2%) and biologists specialized in nutrition (4.7%).

### 3.3 Experiences of weight stigma

**Figure 4:**
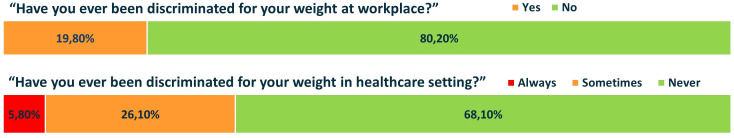
Experiences of obesity-related stigma at workplace and in healthcare setting.

Social stigma and negative attitudes in workplaces have been reported by one out of four patients (19.8%). However, stigma and weight discrimination were also experienced in healthcare settings by a substantial portion of patients (31.9%). Of these, 5.8% stated to feel always stigmatized by healthcare providers (**Figure 4**).

### 3.4 Knowledge and perceptions of obesity treatment options

Most patients (72.1%) felt they were not sufficiently informed about anti-obesity medications and among those who felt well informed about anti-obesity medications, 75% declared that they induce weight loss by reducing hunger and food intake, by limiting absorption of calories (17.5%) or increasing energy expenditure (7.5%). A relevant part of patients’ knowledge derived from healthcare providers (specialists: 46.8%; primary care physicians: 10.9%), followed by social networks (19.1%), friends or acquaintances (11.8%), television (8.6%) and newspapers (2.7%).

**Figure 5:**
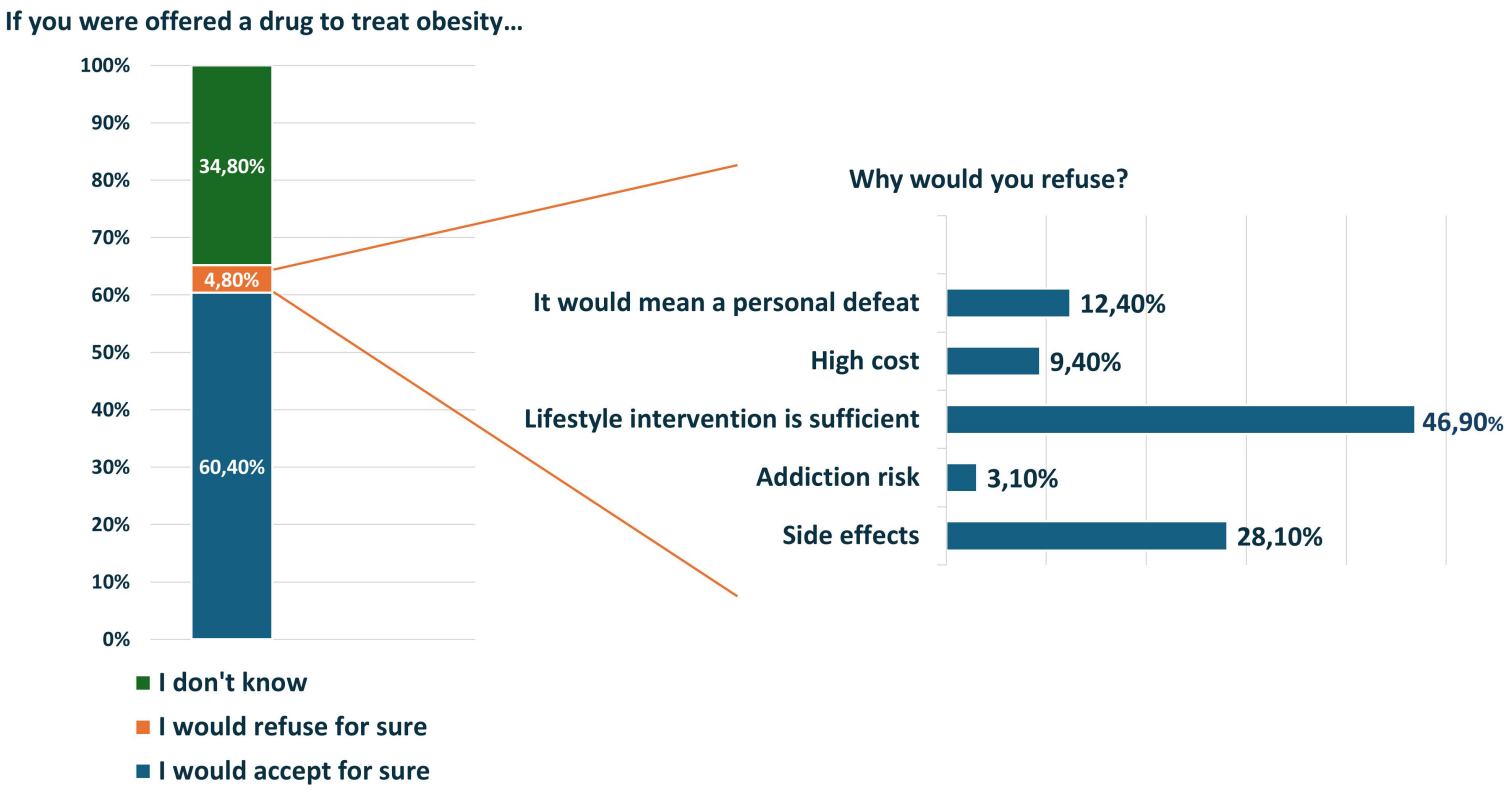
Patients’ perceptions about pharmacologic treatment of obesity.

Weight loss medications were considered useful (63.2%) or necessary (18.4%) by the majority of patients, but only 60.1% would accept without any hesitation a pharmacologic treatment. The remaining considered anti-obesity drugs useless (13.2%) or harmful (5.2%) (**Figure 5**). The main reasons for refusal of pharmacological treatment were the belief that lifestyle intervention is enough to treat obesity (46.9%), the fear of side effects (28.1%) or drug addiction (3.1%), the feeling of ‘defeat’ (12.5%) and high cost (9.4%) (**Figure 5**). Similarly, although a 13.2% of participants considered bariatric surgery dangerous, for most patients it is useful (73.3%) or necessary (13.6%).

Finally, a perception of partially (36.5%) or completely (25.4%) inadequate support from the National Healthcare System emerged in most of the interviews.

## 4 Discussion

In this study, we explored the attitudes towards obesity, self-awareness, misperceptions of weight loss strategies, experiences of weight stigma and educational needs of patients living with overweight or obesity. The survey was completed by 300 participants who for the first time accessed the outpatient services of some of the main hospitals in the Lazio region, central Italy, accredited under the EASO’S Collaborating Centers for Obesity management initiative. The study reflects the “usual” patient that accesses these services, with a prevalence of middle-aged women. Approximately half of these patients came to visit on their own decisions or following the suggestions of relatives or acquaintances, which could mean that obesity is considered a medical issue only by some of the patients. The other half of the patients had been referred by physicians, most by other specialists and only a minority by their primary care providers. In a previous study conducted in our region, it was shown that about two-thirds of primary care providers believed that people have at least some control on their weight (9), thus underestimating their need for support and prescribing specific visits less than necessary. Furthermore, communication about weight is problematic for many clinicians, who cautiously wait for the patient to start talking about obesity (11).

The issue of obesity awareness, stigma and access to all treatment options for people with obesity represents a major obstacle to the implementation of obesity management. There are some studies that have evaluated the problem of awareness of obesity in people suffering from this illness and, to date, there is only one study focusing on obesity awareness among Italian people. In fact, Italy’s results from the ACTION-IO study (10) demonstrated several gaps in terms of perceptions and attitudes towards obesity and obesity care both of people with obesity and healthcare professionals. In general, the results from Italy align with those of the global cohort, indicating that Italian individuals with obesity and healthcare professionals hold attitudes and beliefs similar to their counterparts worldwide (12). Our data support the known difficulties in self-perception of the disease in people suffering from obesity. In a recent publication that has evaluated obesity awareness and its relationship to sociodemographic characteristics (13), a significant relationship between higher educational attainment and obesity awareness has been shown, underlying that obesity awareness is significantly related to socioeconomic status and that the higher the household monthly income, the higher the level of awareness (14). In our study only half of the patients correctly identified themselves as being affected by obesity. The others defined themselves as overweight or normal weight. About 30% of our participants declared to have a university education, half of them completed high school, but 1 in 5 had an education below the high school level. As regards the employment situation, there was a good proportion of unemployed people (> 10%) who inevitably found themselves in conditions of economic difficulty. In Lazio Region the problem of unawareness of obesity has been previously investigated among parents of children with overweight/obesity: almost 50% of mothers of overweight children and 10% of mothers of children with obesity believed that their child was normal weight or underweight and 72% of mothers of overweight children and 45% of mothers of children with obesity believed that their child ate “the right amount” (15). These data, coming from a program for monitoring and promoting healthy weight in childhood and adolescence, promoted by the Ministry of Health, could help explain how the problem of self-perception can also be transferred to the world of adults.

The mistaken belief that obesity is merely a lifestyle choice, which can be reversed by sheer willpower and exercise, has firmly taken hold in the minds of both the general public and a significant portion of the medical community. The issue of self-perception regarding obesity is pervasive and continues to affect the management of the condition today. Our data showed that the majority of patients declared themselves completely responsible for their condition and a good portion of them believe that obesity is always treatable through willpower-based lifestyle interventions. These results are in agreement with those already demonstrated in the ACTION-IO study (10), in which most people with obesity took complete responsibility for their own weight loss and waited considerable time before seeking help from a healthcare professionals.

Although the majority of patients included in our study declared that they had first-degree relatives affected by obesity, only a few of them considered obesity to be a genetic disease. This self-conviction on the part of patients clashes with greater difficulties in managing the disease. A not very recent study showed that people who were told that obesity is not a disease with a genetic predisposition were less likely to follow physicians’ recommendations in terms of diet and physical activity as if the lack of genetic susceptibility freed them from the idea of being sick and being able to be cured. Conversely, people who were told that obesity is also a hereditary disease were more inclined to follow weight loss treatment programs (16). In this study, the poor perception of obesity as a disease with a genetic component means that the majority of participants considered it at least in part only associated with nutritional or psychological problems.

The damaging effects of obesity stigmatization are widespread and include psychological, physical, and socioeconomic harm. There are many studies that have dealt with the stigma of obesity and proposed possible solutions to stem the phenomenon. In our study, social stigma and negative attitudes in workplaces have been reported by one out of four patients and stigma and weight discrimination were also experienced in healthcare settings by a substantial portion of patients. A recent review on weight stigma experienced by patients with obesity in healthcare settings has described the large experience of weight stigma across primary, secondary, and tertiary healthcare settings for patients living with obesity who are victims of both verbal and non-verbal stigmatizing behaviors (17).

Stigma and discrimination against obesity are pervasive and an important consequence is that this limits the possibility to talk and learn about an important medical issue. The majority of patients reported a lack of information related to anti-obesity medications, and among patients who reported being well informed about pharmacological treatments of obesity not all of them knew the main mechanisms of action of these drugs. This might be due to the fact that the information they received did not always come from healthcare providers but also from other less reliable sources such as social networks, friends, television and newspaper which may lead to incorrect knowledge. These results highlight the need to include educational initiatives in obesity management in order to guarantee a complete and correct information of current anti-obesity therapy (8, 10).

Interestingly, data report that weight loss drugs are considered useful and effective by the majority of patients, but only a part of them would accept a pharmacological therapy without any hesitation. The main reasons for refusal of pharmacological treatment were the belief that lifestyle intervention is enough to treat obesity and the fear of side effects, while only a small part refuses it due to the high costs of the drugs. Similarly, although a relevant part of patients considered bariatric surgery dangerous, for most of them it is useful or necessary. These results can be justified by the fact that most Italian patients with obesity do not consider obesity as a chronic disease (10), therefore they tend to consider general improvements in eating habits and physical activity a sufficient and safe strategy for weight management (12), emphasizing the lack of awareness regarding the importance and effectiveness of multidisciplinary approach. On the other hand, this could also suggest that patients consider only more severe forms of obesity, such as those for which bariatric surgery is indicated, as a disease, while less severe forms may be considered as a para-physiologic condition or just an aesthetic problem. This points out an educational need for patients with obesity to have a more adequate knowledge and perception of how obesity deeply impacts on their health and to better understand the most suitable treatment strategies for their needs (10).

Finally, another critical issue is highlighted by the lack of support in obesity care from the National Healthcare System reported by patients, which could lead people with obesity to prefer self-management of their weight or to lack motivation and disinterest in their health condition (12). In Italy the National Healthcare System offers free treatments for many diseases, but not for obesity, and this can sustain the erroneous consideration that obesity is not a disease, nor a public health threat. Therefore, our data underline the need to consider obesity as a public healthcare priority and to act rapidly and synergistically in order to optimize obesity management and to reduce long-term healthcare burden.

This study presents some limitations. First of all, despite the heterogeneity of our participants in terms of age, educational level and employment status, the results of our study may be influenced by its “regional” nature and further studies are needed to extend their validity and applicability beyond our region borders. Moreover, the prevalence of women should be taken into account, as it is probably representative of the patients who accesses outpatient services for the management of obesity but not of the overall population affected by obesity. Another limitation of this study is that previous treatments that could have influenced the attitudes toward obesity, including lifestyle intervention, were not enquired. Finally, self-administered surveys may lead to response bias, as the participants may have not fully understood the questions.

## 5 Conclusions

In conclusion, this study underscores the imperative of enhancing patient education and public awareness to face the challenges posed by obesity. Our findings reveal a pervasive misconception among patients, who often perceive obesity as a mere lifestyle choice that can be overcome through sheer willpower. Additionally, many patients exhibit inadequate self-awareness and hold unclear notions about the causes of their condition. Regrettably, stigma and discrimination persist even within healthcare settings, influencing patients’ behavior. Too often, individuals rely on unreliable sources such as social networks, television, and friends for information. Therefore, initiatives aimed at addressing these misconceptions and providing accurate information about all available therapeutic options are essential. Only through informed decision-making can patients be empowered to effectively adhere to long-term treatment plans. By tackling these misconceptions, reducing stigma, and bridging knowledge gaps, we can significantly improve patient outcomes and cultivate a supportive healthcare environment.

## Data availability statement

The raw data supporting the conclusions of this article will be made available by the corresponding author, without undue reservation, on request.

## Ethics statement

Ethical approval was not required for the studies involving humans because in Italy ethical approval was determined to be non-essential for a study of anonymous nature, based on regulatory standards and precedent. The study complied with all laws and regulations regarding the management of personal information as required by Italy and the European General Data Protection Regulation. The studies were conducted in accordance with the local legislation and institutional requirements. Written informed consent for participation was not required from the participants or the participants’ legal guardians/next of kin in accordance with the national legislation and institutional requirements because no sensitive data was collected.

## Author contributions

LC: Data curation, Formal analysis, Writing – original draft. BR: Conceptualization, Writing – original draft. EC: Conceptualization, Investigation, Writing – review & editing. SM: Conceptualization, Investigation, Writing – review & editing. DT: Conceptualization, Investigation, Writing – original draft. MM: Conceptualization, Investigation, Writing – review & editing. VS: Investigation, Writing – review & editing. MP: Investigation, Writing – review & editing. DC: Conceptualization, Investigation, Writing – original draft. VG: Conceptualization, Data curation, Funding acquisition, Investigation, Methodology, Supervision, Writing – original draft.
